# Procedural Pain Management in Patients with Cerebral Palsy Undergoing Botulinum Toxin Injection: A Systematic Review and Meta-Analysis

**DOI:** 10.3390/toxins17070317

**Published:** 2025-06-22

**Authors:** Silvia Faccioli, Alessandro Ehsani, Shaniko Kaleci, Giulia Tonini, Ilaria Tagliani, Mario Vetrano, Silvia Sassi

**Affiliations:** 1 Pediatric Rehabilitation Unit, Azienda Unità Sanitaria Locale IRCCS of Reggio Emilia, 42122 Reggio Emilia, Italy; silvia.sassi@ausl.re.it; 2 Physical Medicine and Rehabilitation Unit, Sant’Andrea Hospital, Sapienza University of Rome, 00189 Rome, Italy; alessandro.ehsani@studenti.uniroma1.it (A.E.); mario.vetrano@uniroma1.it (M.V.); 3 Surgical Medical and Dental Department of Morphological Sciences Related to Transplant, Oncology and Regenerative Medicine, University of Modena and Reggio Emilia, 41125 Modena, Italy; shaniko.kaleci@unimore.it; 4 Department of Biomedical and Neuromotor Sciences, Alma Mater Studiorum, University of Bologna, 40126 Bologna, Italy; giulia.tonini@studio.unibo.it; 5 Department of Medicine and Surgery, University of Parma, 43126 Parma, Italy; ilaria.tagliani@unipr.it

**Keywords:** cerebral palsy, pain, botulinum toxins, sedation, analgesia, adverse effects, rehabilitation, spasticity

## Abstract

Background: The aim of this systematic review is to investigate effectiveness and safety of sedation–analgesia techniques in controlling pain during botulinum injections in patients with cerebral palsy (CP). Methods: The Pubmed, Cinahl, and Scopus databases were searched. Inclusion criteria were as follows: cerebral palsy; any type of outcome measure regarding pain and side effects assessment; any type of studies; and English language. RoB2 and Robins-I were applied to assess the risk of bias. Tables and forest plots synthetized the findings. Results: Seventeen reports were included; most regarded pain control, and ten investigated side effects. Three were RCTs, three were controlled, and twelve were observational studies. Several techniques were used, often in combination, such as non-pharmacological approaches (clown care or virtual reality); topical anesthesia with Emla^®®^, vapocoolant spray, or ice; and light-to-deep sedation with inhaled nitrous oxide, intranasal fentanyl, rectal, enteral, or intravenous midazolam, or intravenous ketamine or propofol. Vomiting and oxygen desaturation were uncommon complications. Conversely, the pooled incidence of other minor side effects was 6.39% (95% CI: 1.47–14.42%) under the random-effects model, with considerable heterogeneity. Conclusions: All the techniques are safe, if administered in an appropriate setting. Deep sedation is more effective in pain control but requires an anesthetist. A combined individualized approach is preferrable. PROSPERO CRD42025639999.

## 1. Introduction

The International Association for the Study of Pain (IASP) defines pain as an “unpleasant sensory and emotional experience associated with, or resembling that associated with, actual or potential tissue damage.” The new definition, revised in 2020 from the previous one from 1979, confirms that pain is an entirely subjective experience, in which purely sensory components (nociception related to the transfer of the pain stimulus from the periphery to central structures) and experiential and emotional components converge, significantly modulating what is perceived. The revision of the definition includes some important specifications emphasizing that pain is multimodal, always due to a combination of biological, psychological, and social factors; is not just nociception, so it cannot be inferred solely from neurosensory activity; has negative effects on functionality and overall well-being when it becomes chronic, thus losing its adaptive role; and can also be present in patients unable to communicate and therefore unable to describe their experience. This definition, enriched with ethical aspects, is very important for vulnerable populations, for the so-called “voiceless” individuals, newborns, elderly, and children with neurological, intellectual, or motor disabilities, such as children with cerebral palsy (CP).

Persons with CP may have difficulty in self-reporting pain, and various studies [1,2,3,4] have shown that children receive analgesia less often than adults for the treatment of their pain, even though untreated acute pain could lead to long-term negative effects in children [5], and anxiety and fear could lead to the failure of the procedure itself [6].

In children requiring repeated painful procedures, the pain experienced during the initial procedure has a significant influence on pain and anxiety scores in subsequent procedures [7] and can reduce the effect of painkillers over time [8]. Therefore, in addition to pain treatment, sedation potentially plays an important role in adequately relieving anxiety and fear in children.

Some medical procedures are recognized as possible causes of pain for this population, including botulinum toxin injections.

Intramuscular botulinum toxin is a treatment for spastic hypertonia in adult and pediatric patients with CP [9]. The benefits and safety of injections have been extensively studied [10]. To optimize effectiveness and reduce collateral effects, localization of the involved muscles should be performed with electromyographic needle guidance and/or ultrasound. In most cases, these injections are repeated several times during the child’s growth to prevent secondary orthopedic complications related to spasticity [11]. The treatment is generally well tolerated [12], but children, adolescents, and their parents may be concerned about the pain associated with the procedure [13]. The pain caused by the injection might induce long-term anxiety and has been reported as a reason for abandoning the treatment [14].

Internationally, most teams perform toxin injections under short-acting general anesthesia (GA) [15]. However, these approaches require venous access for drug administration and patient safety, which is itself painful, and deeper sedation exposes the child to a higher risk of complications. Therefore, in a cost–benefit balance, they may be disadvantageous, especially considering that children with CP are at greater risk of anesthetic complications compared to healthy children [16]. In the literature, side effects of GA are reported in a percentage ranging from 7.7% to 21.7%, although they are not severe and are reversible within 24 h [17,18]. Subjects with CP present multisystem-associated comorbidities (i.e., chest wall deformity and restrictive lung disease, upper airway obstruction, swallowing dysfunction, gastroesophageal reflux, inefficient cough, sleep-disordered breathing, epilepsy) that may present perioperative challenges for anesthesia [19,20,21,22]. Therefore, alternative sedation–analgesia techniques to GA should be considered when performing botulinum toxin injections in these patients. The increased use of botulinum toxin and the growing number of studies evaluating its efficacy and safety over the past 40 years contrast with the absence of established guidelines for pain control during botulinum injections [23,24,25]. Friedrichsdorf et al. recommended a multimodal approach to address the prevention and treatment of acute and chronic pain in children, including pain caused by needles [26]. Krauss et al. reviewed the indications, the pharmacology, and adverse effects of drugs for pediatric procedural sedation–analgesia in general [27]. Nonetheless, the specificity of botulinum toxin injection procedures in patients with CP requires a proper and updated approach [28].

The aim of this systematic review is to investigate effectiveness and safety of the various techniques of sedation–analgesia to control procedural pain during botulinum toxin injections in patients with CP.

## 2. Results

**Figure 1:**
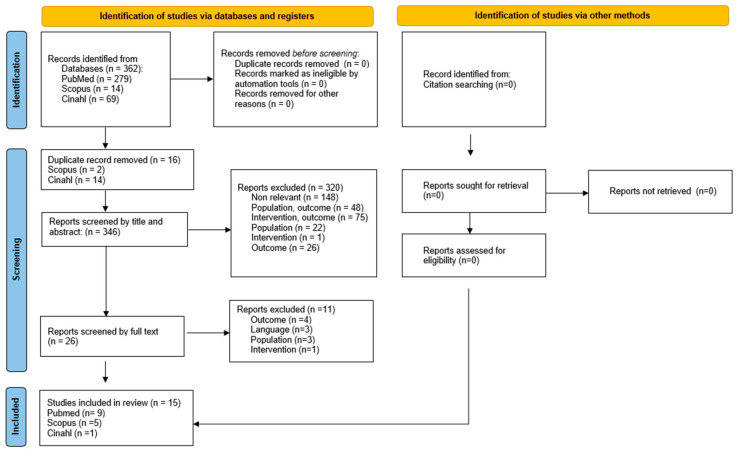
PRISMA flow diagram relative to PICO 1.

**Figure 2:**
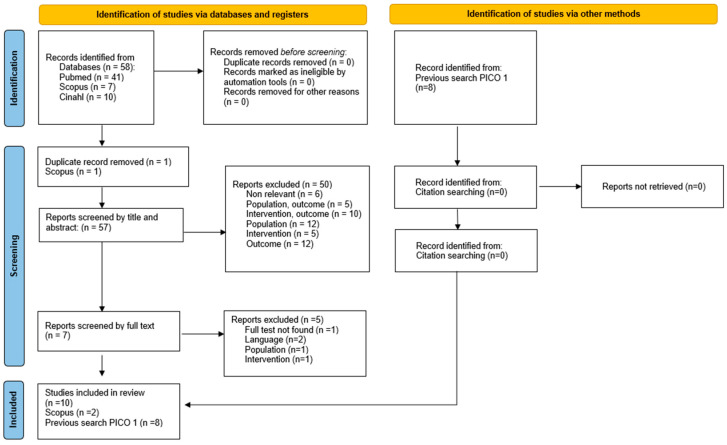
PRISMA flow diagram relative to PICO 2.

After full-text analysis, seventeen studies were finally included in the review [29,30,31,32,33,34,35,36,37,38,39,40,41,42,43,44,45], and one reported the results of two different audits [36]. Fourteen studies focused on procedural pain control [29,30,31,32,33,34,35,36,37,38,41,42,43,45], and seven of them also reported data regarding adverse events [29,30,35,36,37,38,43]. Only three studies investigated side effects as primary or secondary outcomes [39,40,44]. Therefore, fifteen studies were included relative to PICO 1, and ten were finally considered for PICO 2. Figure 1 and Figure 2 provide details on the identification and selection (PRISMA flow diagram) of studies related to PICO 1 (effectiveness in pain control) and 2 (safety of sedation analgesia approach), respectively.

### 2.1. Risk of Bias Assessment

An overall synthesis of RoB assessment of the included studies is presented in Figures S1 and S2 by means of RoB2 [46] for crossover studies and an individually randomized parallel group (IRPG) trial, respectively; in Figure S3 by means of ROBINS-I [47] for non-randomized controlled studies; and in Table S1 by means of the JBI tool [48] for case series. Samples including a wide age range, mixed gross motor functioning levels, mixed diagnosis, and combined interventions were considered confounding factors. Furthermore, a lack of a time interval between injections and assessment in different conditions was included as a possible confounding aspect because of the possible influence of the painful experience on the subsequent procedure.

### 2.2. Evidence Synthesis

Several types of sedation and analgesia protocols were used in the included studies, such as a combination of inhaled nitrous oxide (N₂O) and Emla^®®^ [35,37,41]; inhaled nitrous oxide (N₂O) and Emla^®^ versus general anesthesia [38]; Emla^®^ versus Gebauer Pain Ease vapocoolant spray versus ice [31,42]; a combination of intranasal fentanyl and topical anesthetic cream versus previous sedation [36]; midazolam versus a combination of midazolam and ketamine (MIKE) [36]; a combination of rectal midazolam and ketamine [39,40]; Entonox (self-administered fixed 50% nitrous oxide/oxygen) versus enteral midazolam with Emla^®^ [30]; inhaled nitrous oxide (N₂O) versus enteral midazolam [29]; a combination of propofol and ketamine [44]; and inhaled nitrous oxide (N₂O) versus deep intravenous sedation or light sedation with midazolam [32]. Furthermore, different types of non-pharmacological strategies (behavioral interventions) were proposed, such as clown care [33]; virtual reality [43]; and medical clowns versus usual distractions (music, television, video games). All studies used botulinum toxin type A (BTX-A).

To assess pain, several validated outcome measures were used in the included studies, such as visual analogue (VAS); Children’s Hospital of Eastern Ontario Pain Scale (CHEOPS); faces pain acale (FPS); Face, Legs, Activity, Cry, Consolability (FLACC); Numeric Pain Rating Scale (NRS); Wong–Baker faces scale (WBFS); and Color Analogue Scale (CAS). In a study evaluating the effectiveness of BART (biofeedback-assisted relaxation training), fear was also considered and assessed using the CFS (Children’s Fear Scale). Parent satisfaction with the procedures, carer perceptions of their child’s pain, safety, and efficacy were also assessed but using non-validated tools [36,38,43].

The studied populations ranged from a minimum of 30 to a maximum of 171 participants. Most studies focused on patients with cerebral palsy (CP) and related issues such as spasticity and muscle pain (1347 patients in total). A few reports included patients affected by other pathologies, such as 4 subjects with acquired brain injury or hereditary spastic paraplegia [34]; 30 patients presenting spasticity at the ankle [31] and 1 at the adductors [37]; 60 other neurologic and musculoskeletal conditions [42]; 1 spinal cord injury and 1 pontine hemorrhage [43]; and 12 cognitive disorders [45]. The average age of participants varied between 5 and 7 years, with standard deviations suggesting a fairly wide distribution. Only the study by Fung et al. [31] investigated patients older than 18 years (30 subjects, age 55 ± 16).

**Figure 3:**
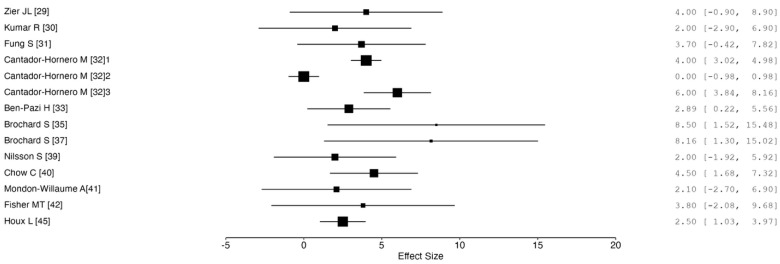
Forest plot showing the mean pain of the included studies; ^1^ group II: N₂O; ^2^ group III: deep iv sedation; ^3^ group IV: oral or rectal BDZ.

Twelve studies reported data regarding mean procedural pain while applying sedation or analgesia techniques by means of comparable outcomes measures. A synthesis of these results is represented in Figure 3. Optimal pain control was reached through deep intravenous sedation in group III of the study by Cantador H.M. et al. [32]. By means of all the other techniques (N₂O, topical anesthesia, BDZ, VR experience, clowns), the mean pain was between 2 and 8.5 over a 0–10 scale [29,30,31,32,33,35,37,39,41,42,43,45].

Detailed characteristics of the included controlled studies and case series are represented in Table 1 and Table 2, respectively. The outcome measures are described, statistically significant results are reported in bold, and the level of significance was 5% for all studies (*p* < 0.05). Tables include data relative to both PICO 1 and 2; side effects are reported in a separate column.

Fung S et al. [31] conducted a randomized crossover study comparing Emla^®^, vapocoolant spray, and ice during consecutive BTX-A injections in the gastrocnemius muscle. The gastrocnemius muscle was divided into four quadrants, and one of the three different anesthetic agents was applied to each quadrant, with one quadrant using no anesthetic as a control. The authors suggested that Emla^®^ and ice were comparable and significantly more effective than the control and vapocoolant spray, but ice might be a more convenient option because of the brief application time (compared with Emla^®^). On the contrary, vapocoolant spray might have made the patients more sensitive to pain. Nonetheless, the results were limited by the high risk of bias arising from carryover effects due to the lack of time interval between injections and, consequently, a possible influence of the painful experience on the subsequent procedure.

Ostojic K et al. [34] compared biofeedback-assisted relaxation therapy (BART) with distraction therapy in an RCT using the FPS scale as a pain outcome measure. The authors concluded that no significant differences were observed in overall pain and worst pain between BART therapy and distraction therapy. Individual factors, such as pre-procedural anxiety and younger age, were associated with worse outcomes in terms of pain, fear, and anxiety. “Some concern” was attributed to risk of bias because it is unclear whether the randomization sequence was fully concealed, and some participants dropped out of the study without reported reasons.

The RCT by Zier et al. [29] compared the effectiveness of nitrous oxide (N₂O) and rectal midazolam by means of the FLACC scale. It concluded that pain was significantly lower in children who received nitrous oxide, while the level of sedation at discharge was significantly higher in the midazolam group. Time spent in the clinic did not differ significantly between the two groups, but the N₂O group showed a quicker return to wakefulness after the procedure, and parents in the N₂O group reported a better experience. The overall risk of bias assessment presented “some concern” due to the lack of reference to a previously presented study protocol, leaving room for possible changes in analysis.

Kumar et al. [30] compared the effectiveness of nitrous oxide with orally administered midazolam in an RCT, reporting a lower mean pain score with nitrous oxide than with midazolam. Nonetheless, the study group had a significantly different mean age compared to the control group (3 years vs. 9 years), introducing a potential confounding factor that led to a “critical” risk of bias level.

Cantador Hornero et al. [32] published a non-randomized controlled trial comparing various sedation–analgesia strategies, such as Emla^®^, nitrous oxide, deep sedation, and mild sedation with midazolam. Pain was assessed by means of the WBS and FLACC scales. The authors concluded that deep sedation was more effective for pain control with higher parental satisfaction, and Emla^®^ was effective in 50% of cases. Nonetheless, the group assignment was previously defined based on the injection sites (number and depth of intramuscular injection), the level of cooperation of the child, and the available resources. This led to a significant age disparity between the groups and a critical risk of bias level.

In the quasi-randomized controlled trial by Ben Pazi et al. [33], clown care was compared to a standard approach (cooling with ethyl chloride), and pain was assessed by means of the VAS scale. The authors reported that the presence of clowns during procedures significantly reduced the pain perceived by children compared to the control group. A positive effect of clown care was also observed during subsequent procedures without clowns, suggesting a carryover effect. Nonetheless, severe mental retardation, autistic spectrum disorders, or severe anxiety requiring general anesthesia were exclusion criteria. Furthermore, the absence of an explicit strategy for handling missing data relative to dropouts led to a “moderate” risk of bias.

Both studies by Brochard et al. [35,37] were prospective observational studies based on data acquired during an evaluation of professional practice. Both investigated the effectiveness in terms of pain relief of nitrous oxide combined with Emla^®^ by means of the CHEOPS scale. The first study by Brochard et al. [35] demonstrated methodological robustness, but the participant selection was not entirely consecutive, potentially introducing selection bias. The study concluded that the protocol was effective in 50% of patients. Brochard et al. [37] reported that analgesia was effective in 62% of cases but presented some concerns regarding the participant selection (half of the sample was derived from the previous study), which was not entirely transparent. This could have influenced the sample’s representativeness and, consequently, the generalizability of the results. Most participants had a low level of cognitive disability and were undergoing their first injection experience.

Gubbay et al. [36] published data from two audits. One assessed parental satisfaction by comparing midazolam with intravenous midazolam and ketamine combination (MIKE). It concluded that both procedures were well tolerated, with a percentage of parents who previously experienced only midazolam reporting better tolerance with the MIKE procedure. Only transient respiratory distress was reported as side effect. The second audit [36] compared the effectiveness and safety of intranasal fentanyl combined with Emla^®^ to previous methods. Both presented several limitations due to several missing data (i.e., inclusion criteria and sample characteristics were not specified, it was unclear if participant selection was consecutive, and the analysis was only descriptive).

The retrospective study by Fisher et al. [42] investigated the predictive factors on procedural and post-procedural pain, comparing Emla^®^ with vapocoolant spray by means of the NRS and WBS scales. The demographic information was detailed and the statistical analysis was adequate, but the selection was unspecified regarding consecutiveness and possibly introduced bias in terms of overestimating or underestimating the effectiveness and safety of the interventions due to unaccounted-for variability among participants. The authors concluded that topical anesthesia and injections in hands, legs, and adductors were positive predictors for procedural pain, while age was a negative predictor. For post-procedural pain, age became a positive predictor, as did procedural pain.

Forrester et al. [38] compared nitrous oxide combined with Emla^®^ to general anesthesia, concluding that general anesthesia was more effective for pain control according to parents but required more specialized personnel. Nonetheless, the study presented serious methodological limitations, such as a lack of clear inclusion criteria, non-detailed clinical information, non-standardized and reproducible evaluation scales, and no specification as to whether the patients were consecutively included or selectively chosen. The statistical analysis was poorly structured, reducing the robustness of the results.

The remaining observational studies investigated procedural pain by means of the FLACC scale while applying nitrous oxide combined with Emla^®^ [41], immersive virtual reality [43], and clown care [45]. They all presented methodological limitations, in particular relative to the selection of participants, limiting solid conclusions. Nilsson et al. [39] investigated the effectiveness and safety of rectal midazolam combined with intravenous ketamine in a prospective observational study using the FLACC and CAS scales. The overall quality was good except for minimal concerns relative to participant selection and transparency of inclusion criteria. The authors concluded that there was a negative GMFCS/pain relationship and a significant difference between outcomes reported by medical staff compared to parents, with parents reporting higher pain scores.

Only two the retrospective observational studies exclusively assessed the safety of intravenous ketamine combined with midazolam [40] or propofol [44]; the overall methodological quality was good except for bias relative to participant selection.

#### Side Effects

Side effects were reported in the study by Brochard et al. [35], in which the authors declared that three children vomited after the session with no other complications, and two children had particularly vivid dreams in the nitrous oxide–Emla^®^ combination. In the study by Gubbay et al. [36], the authors observed one minor adverse effect involving transient distress after the MIKE procedure (midazolam + ketamine). Nilsson et al. S [39] observed nausea in 21.7% of cases, pain in 8.9% of cases, and sleep disturbance in 7.7% of cases when using a combination of rectal midazolam and ketamine. Zier et al. [29] observed desaturation < 92% in one case after rectal midazolam, while in the inhaled nitrous oxide (N₂O) group, one patient experienced nausea, one experienced headache and pallor, four experienced vomiting, and two experienced desaturation < 92%. Chow et al. [40] reported four cases of rash, three cases of nausea and vomiting, one case of limb tremors, one case of mild headache, one case of nightmares on the evening of the procedure over 87 children treated with a combination of intravenous ketamine and midazolam. In the study by Louer et al. [44], adverse events were reported in 10.1% of procedures involving propofol plus ketamine (35 out of 345 procedures), with hypoxemia occurring in 9.6% (33 procedures) and transient apnea in 1.4% (5 procedures). In general, no serious adverse events occurred.

### 2.3. Meta-Analysis

**Figure 4:**
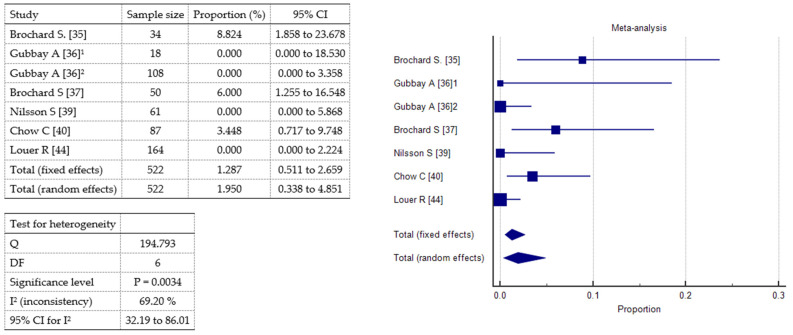
Forest plot showing the meta-analysis of the incidence of vomiting;^1^ 1^st^ audit; ^2^ 2^nd^ audit.

**Figure 5:**
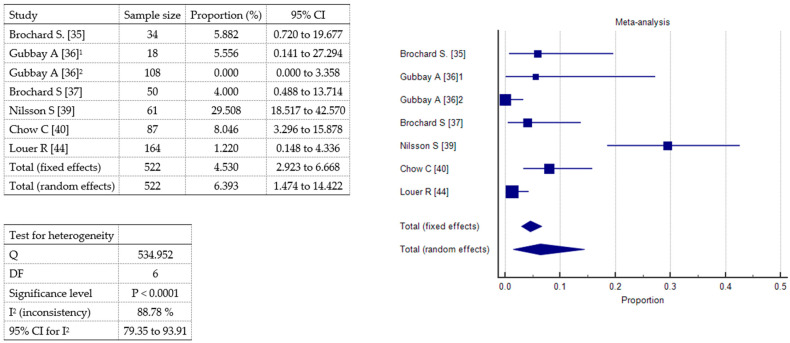
Forest plot showing the meta-analysis of the incidence of mild adverse effects, i.e., headache or nausea;^1^ 1^st^ audit; ^2^ 2^nd^ audit.

**Figure 6:**
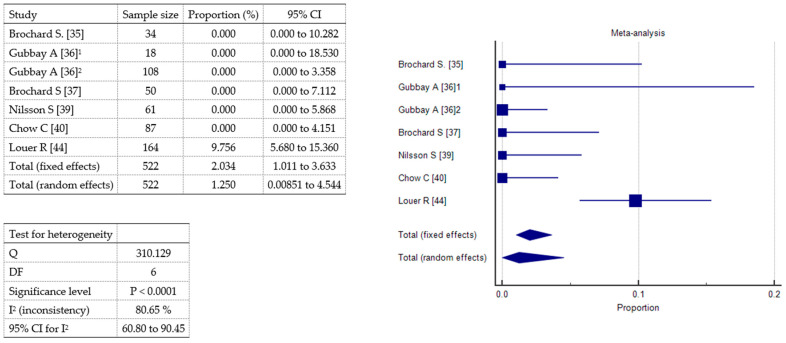
Forest plot showing the meta-analysis of the incidence of oxygen desaturation events;^1^ 1^st^ audit; ^2^ 2^nd^ audit.

A meta-analysis was conducted to evaluate the incidence of three specific adverse events vomiting, other mild side effects (including headache and nausea), and oxygen desaturation associated with sedative or analgesic interventions in pediatric patients. A total of seven studies were included, comprising 522 patients. Vomiting (Figure 4) was reported in all included studies. The pooled incidence under the fixed-effects model was 1.29% (95% CI: 0.51–2.66%), while the random-effects model yielded a slightly higher estimate of 1.95% (95% CI: 0.34–4.85%). Statistically significant heterogeneity was observed (Q = 19.48, df = 6, *p* = 0.0034), with an I^2^ value of 69.2% (95% CI: 32.19–86.01%), indicating moderate-to-substantial inconsistency among studies. Other mild adverse effects (Figure 5), including headache and nausea, were also reported across all seven studies. The pooled incidence was 4.53% (95% CI: 2.92–6.67%) under the fixed-effects model and 6.39% (95% CI: 1.47–14.42%) under the random-effects model. Heterogeneity was considerable (Q = 53.50, df = 6, *p* < 0.0001), with a high I^2^ of 88.78% (95% CI: 79.35–93.91%). Oxygen desaturation events (Figure 6) were documented in all studies. The fixed-effects model estimated a pooled incidence of 2.03% (95% CI: 1.01–3.63%), whereas the random-effects model indicated a slightly lower incidence of 1.25% (95% CI: 0.0085–4.54%). Heterogeneity was statistically significant (Q = 31.01, df = 6, *p* < 0.0001), with an I^2^ of 80.65% (95% CI: 60.80–90.45%). These findings suggest that vomiting and oxygen desaturation are relatively uncommon complications of pediatric sedation and analgesia, while other mild adverse effects occur more frequently. However, the substantial heterogeneity observed across all outcomes underscores variability in study design, patient characteristics, and definitions of adverse events.

## 3. Discussion

The main objective of this review was to assess the effectiveness and safety of sedation–analgesia interventions to reduce procedural pain. The studies were heterogeneous in terms of study type, population, pain assessment, and sedation–analgesia approach.

The analyzed studies included crossover and parallel RCTs (randomized controlled trials), as well as controlled prospective and retrospective studies and case series. Most studies focused on pediatric patients with cerebral palsy, except one [31]. The age range was generally wide in most studies, extending from children to young adults; this was considered as an RoB. The pain assessment tools varied between studies (VAS, CHEOPS, Wong–Baker FACES, FLACC), including non-validated outcome measures and making direct comparison difficult. Additionally, some studies did not include adequate control groups or sufficiently large samples to ensure generalizable results.

The interventions included the use of analgesic gas (nitrous oxide), non-pharmacological approaches (clown care, virtual reality, BART, usual distraction techniques), local anesthetics (Emla^®^, ice, vapocoolants spray), and light and deep sedation (midazolam, ketamine, intranasal fentanyl, propofol). These techniques were used alternatively or in some cases in combination. The non-pharmacological approach using clown therapy, BART, and virtual reality [33,34,43] has shown benefits in improving the patient experience, reducing discomfort or anxiety related to sedation (e.g., the facial mask), avoiding the need for fasting, and reducing recovery times, especially by preventing the side effects of sedation. However, these interventions do not appear to be more effective than usual distraction therapy (such as videos or music), nor are they a substitute for pharmacological approaches. The use of sedation techniques with ketamine [40] or combined treatment with propofol and ketamine [44] has proven successful in pain control, with a low adverse event rate (6.6% and 10,1%, respectively). However, patients require at least 2 h of monitoring after the procedure and more specialized personnel [36]. Additionally, these approaches require venous access for drug administration and patient safety, a procedure that is itself painful. Moreover, deeper sedation exposes the child to a higher risk of complications, which may make it disadvantageous in a cost–benefit balance. This is particularly relevant considering that children with cerebral palsy (CP) are at higher risk of anesthetic complications compared to healthy children. As an alternative to deep sedation, the most frequent approach was nitrous oxide combined with local anesthetic cream [29,35,36]. Significant effectiveness in reducing procedural pain was observed in one out of two children, suggesting that this protocol should be a first choice. Only non-serious transient side effects were reported. Nonetheless, it implies organizational constraints related to its application and the necessary waiting time (about 1 h for local anesthetic cream). The pooled analysis regarding mean pain reduction showed a statistically significant reduction in pain under the fixed-effects model (SMD = −0.366, 95% CI: −0.575 to −0.157; *p* = 0.001), indicating a modest benefit of sedation or analgesia over the control. However, this result was not statistically significant under the random-effects model (SMD = −0.990, 95% CI: −2.196 to 0.216; *p* = 0.107),due to very high heterogeneity (I^2^ = 96.85%). The variability in pain outcomes likely reflects differences in pain assessment tools, timing of evaluation, and sedation protocols. Some studies may have employed procedures associated with differing levels of baseline discomfort, further contributing to heterogeneity. It should also be noted that considering the average pain as the outcome may not be sufficiently “protective” for the patients. It would be better to consider the proportion of patients with pain below a certain threshold of pain (e.g., 3), as proposed by a few of the included studies as a secondary outcome [32,41].

Only transient side effects were reported, mostly resolved with supplemental oxygen. Only Louer et al. [44] provided a clear definition of what were considered side effects, what were considered serious side effects, and, additionally, a clear definition of procedure time, recovery time, and total time, which are all fundamental data in defining the efficacy and safety of an anesthetic procedure. Adverse events were defined as development of transient hypoxemia (oxygen desaturation of less than 90% for 30 seconds), hypotension (drop in systolic blood pressure below the expected age-appropriate normal range or dropping by 20% from starting systolic blood pressure), transient apnea, nausea, and vomiting. Serious adverse events were defined as endotracheal intubation, respiratory or cardiac arrest, failure to complete the procedure, and transfer to higher-level care. Procedure time was defined as the time between the first dose of propofol until the procedure was completed. Recovery time was defined as the interval between the end of the procedure until the patient’s level of consciousness returned to Ramsay level 2 [49]. Total time was defined as the time, recorded by the assisting nurse, from the first administration of the sedation–analgesia drug until the patient was ready for discharge. The lack of such information in other studies was considered a risk of bias. In general, we do not have substantial evidence to link the reported side effects to the sedation–analgesia procedure or the botulinum toxin injection. Nonetheless, we can state that none of the reported side effects are listed among those commonly found in the literature for botulinum toxin, except for respiratory issues, which are unlikely to appear within the first minutes after injection if caused by the toxin, as they would require more time to manifest. Therefore, we can hypothesize that most of the observed side effects were probably due to the sedation–analgesia procedures.

The meta-analysis demonstrated that vomiting and oxygen desaturation were relatively rare, with pooled incidences below 2% in both the fixed- and random-effects models. In contrast, other mild adverse effects, such as headache and nausea, were more commonly reported, with a pooled incidence of approximately 6% under the random-effects model. These findings are consistent with prior research suggesting that while serious adverse events in pediatric sedation are uncommon, mild side effects remain relatively frequent and should be considered in clinical decision-making. However, significant heterogeneity was observed across all outcomes, particularly for mild adverse effects (I^2^ = 88.78%) and oxygen desaturation (I^2^ = 80.65%). At a glance, two studies differed from the others. Nilsson S. et al. [39] reported the highest incidence of mild side effects, such as nausea or sleep disturbance, and Louer R. et al. [44] reported the highest incidence of desaturation. Ketamine with midazolam [39] or propofol [44] was used in these cases. Differences in drug types, dosages, routes of administration, monitoring protocols, and definitions of adverse events likely contributed to the variability. These discrepancies highlight the need for standardized methodologies and reporting guidelines in future pediatric sedation trials.

These findings have important clinical implications. While pediatric sedation appears generally safe with regard to serious adverse events, clinicians should remain vigilant for mild but bothersome side effects that can affect patient comfort and satisfaction. Furthermore, although pain control was achieved in many cases, the inconsistency across studies calls for more rigorous and uniform evaluation of analgesic efficacy. Both aspects must be considered by clinicians when choosing the sedation–analgesia approach. A combined step-by-step approach might be the best solution, starting with topical anesthetic cream and individualized distraction techniques. Further sedation (e.g., N₂O and/or BDZ) should be provided based on the individual characteristics and the requirements of the injection procedure. Pain is a subjective experience, and patients may vary their pain tolerance based on their current psycho-emotional state. Therefore, clinicians need to individually and currently decide which is the most appropriate approach, balancing the risk of experiencing mild side effects or pain for the best compliance to the ongoing procedure in order to ensure adherence to spasticity treatments repeated over time.

Controversial data were found regarding the correlation between pain and factors such as age, sex, injection site, and number of injections. No correlation was found by Brochard et al. [37]. On the contrary, Cantador Hornero et al. [32] reported a significant association between younger ages and higher pain values, but the sample was too limited to draw conclusions and the results might be influenced by a selection bias: the level of sedation was selected based on the injection sites (number of injections and depth of intramuscular injection), the level of cooperation of the child, and the available resources.

Although none of the studies specifically addressed this topic, we find it appropriate to specify that all sedation procedures, whether mild or deep, must be performed in a protected hospital environment and by trained personnel. Despite being reported at low percentages, the management of sedation-related side effects must be entrusted to physicians and anesthetists who can provide first aid or resuscitation assistance in certain cases, as defined by the pediatric anesthetists guidelines of the Italian Society of Anesthesia, Analgesia, Resuscitation, and Intensive Care (SIAARTI) [50,51]. Additionally, deep sedation via the administration of ketamine and propofol must be strictly reserved for anesthetists.

### Limitations

Several limitations should be acknowledged. First, the number of included studies was relatively small, which may limit the generalizability of the findings. Second, the wide range of sedative and analgesic agents used across studies makes it difficult to draw specific conclusions about any individual intervention. Third, data on patient characteristics (e.g., age, weight, baseline health status) and procedure types were inconsistently reported, which limited the ability to perform subgroup analyses. Finally, the presence of publication bias cannot be ruled out, although this was not formally assessed due to the small number of studies.

## 4. Conclusions

The added value of this meta-analysis is to show that sedation and analgesia in pediatric patients are generally safe, with a low incidence of vomiting and oxygen desaturation. Mild adverse effects are more common, should be anticipated, and should be taken into account when choosing the sedation–analgesia approach.

The variability in pain control efficacy and adverse event rates highlights the need for standardized protocols and outcome measures in future studies. Greater consistency in study design will be crucial to advancing evidence-based sedation practices in pediatric care.

Side effects are usually transient but require expert management. Therefore, trained personnel, including an anesthetist for deep sedation, and a hospital environment are necessary to perform pharmacological sedation–analgesia approaches.

## 5. Materials and Methods

The present study is a systematic review of primary studies performed according to the reporting guidelines of the PRISMA statement [52] and Cochrane’s methodological recommendation [53]. The review protocol was recorded on the PROSPERO public on-line register (CRD42025639999). The study was conducted according to the pre-specified protocol. The scope of the systematic review was structured according to the PICO (Patients, Intervention, Control, Outcome) framework for intervention:

- PICO1
  – P: population with cerebral palsy undergoing botulinum toxin injections.
  – I: procedural pain control techniques.
  – C: no treatment or any other treatment.
  – O: procedural pain.
- PICO2
  – P: population with cerebral palsy undergoing botulinum toxin injections.
  – I: procedural pain control techniques.
  – C: no treatment or any other treatment.
  – O: adverse effects.

A literature search was performed on December 2024 in the following databases: PubMed, Scopus, and Cinahl. The search strategy is reported in Table S2. Articles were searched, with no limit relative to the year of publication and age. On the contrary, a limit was introduced regarding the language (only English) and the type of study, excluding systematic reviews, guidelines, and animal studies. According to these inclusion and exclusion criteria, all studies were screened first by title/abstract and then by full text by two independent authors (AE, GT). Any disagreement was resolved through discussion among authors. Non-retrieved papers and ongoing studies were just recorded as not retrieved.

Two authors independently completed the data extraction (AE, SF, GT, IT). The authors extracted data about the study design and methodology, participant characteristics, protocol details, outcome measures, and the results of the studies. Any disagreement among the authors was discussed and resolved by consensus.

The risk of bias (RoB) was assessed with a domain-based approach using version 2 of the Cochrane risk of bias (ROB2) tool for RCTs (specific for crossover design and IRPG) [46], using the Risk of Bias in Non-randomized Studies of Interventions (ROBINS-I) tool in controlled studies [47], and using the JBI tool for case series studies [48]. The following thresholds were considered to define the overall quality: <70% YES as “critical”, 70–80% YES as “fair”, >80% YES as “good”. The overall judgment based on the RoB and quality of studies is represented through the “Traffic Light” tab in Table 1 and Table 2 (first column). Two independent reviewers (AE, GT) assessed the methodological quality and the risk of bias of all the included studies. Any disagreement was resolved through discussion among the authors. The assessment of RoB did not provide criteria for excluding articles but for stratifying them.

A meta-analysis was conducted to evaluate the incidence of adverse events associated with sedative or analgesic interventions in pediatric patients.

For each study, the number of adverse events and the total sample size were extracted. Incidence proportions and their corresponding 95% confidence intervals (CIs) were calculated. Pooled estimates were computed using both fixed-effects and random-effects models (DerSimonian–Laird method). Heterogeneity among studies was assessed using Cochran’s Q test, with statistical significance set at *p* < 0.05. The I^2^ statistic was used to quantify inconsistency, with values above 50% indicating substantial heterogeneity. Analyses were performed using the MedCalc Statistical Software version 14.8.1 (MedCalc Software bvba, Ostend, Belgium) [54].

## Figures and Tables

**Table 1:** Characteristics of included randomized and non-randomized controlled trials.

Reference	Study Type	Population *	Diagnosis	Intervention	Outcome	Results *^#^	Side Effects
Zier JL [29] 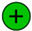	RCT double-blind	50 (29 M, 21 F); mean age 8 y 2 mo ± 4 y 5 mo	CP: DP 11, TrP 3, QP 16, HP 16, other 3; GMFCS I 4, II 24, III 4, IV 13, V 5	Rectal midazolam vs. N₂O (operator titrated 70% maximum nitrous oxide⁄oxygen mixture)	Pain by means of FLACC assessed by parents and nurse. Parents comparation with prior sedation for similar procedures. Sedation levels at discharge.	**Median FLACC: N₂O 4** (0–10); midazolam 6 (0–10). **N₂O better in parental comparison to prior sedation experience.** **Sedation levels at discharge higher in midazolam:** 0 in 3 children, 1 in 10 children, 2 in 9 children, 3 in 3 children, 4 none; in N₂O: 0 in 17 children, 1 in 7 children, 2 in 1 child, 3 and 4 none.	Midazolam: 1 desaturation < 92%. N₂O: 1 nausea, 1 headache and pallor, 4 vomit, 2 desaturation < 92%.
Kumar R [30] 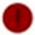	Non-randomized controlled trial	33 (21 M, 12 F); mean age 8 y 1 mo ± 4 y 11 mo	CP: GMFCS I 11, II 6, III 6, IV 5, V 5; HP 8, DP 11, QP 7, other 7	Entonox^®^ (N₂O: 50% nitrous oxide⁄oxygen) vs. enteral midazolam with Emla^®^	Pain measured by means of FLACC, FPS, or VAS (range 0–10). Parents satisfaction.	Median pain scores: midazolam 3, Entonox^®^ 2. Children with pain scores > 5: midazolam 4, Entonox^®^ 3. Parent satisfaction: midazolam 13 satisfied, 0 not satisfied, 3 unknown; Entonox^®^ 18 satisfied, 2 not satisfied, 4 unknown.	None
Fung S [31] 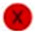	RCT single-blind	30; mean age 55 y ± 16 y	Ankle spasticity	3 anesthetics (Emla^®^, Gebauer Pain Ease vapocoolant spray, ice) compared with no anesthetic (controls)	Mean pain: NRS (range 0–7) and the FPS (range 0–6).	**Emla^®^: NRS 2.20 ± 2.10**, FPS 2.10 ± 2.00; Spray: NRS 3.70 ± 2.40, FPS 3.60 ± 2.50; **Ice: NRS 2.20 ± 2.20, FPS 1.80 ± 2.20;** Controls: NRS 3.70 ± 1.95, FPS 3.07 ± 1.89.	/
Cantador Hornero M [32] 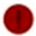	Cross-sectional study	124; mean age 6.75 y	CP: QP 17, DP 66, HP 41 GMFCS I 41, II 35, III 28, IV 8, V 12	Group I, without sedation or topical anesthetic cream; group II, N₂O; group III, deep iv sedation; group IV, light sedation oral or rectal benzodiazepines	Pain and percentage of patients who experienced pain ≤ 2 assessed by means of FLACC < 3 y, WBS 3–7 y, VNS > 8 y (range 0–10). Secondary outcome measures: associations between the level of pain and different variables (GMFCS score, dose of BoNTA, number of injections, MAS score, functional classification of CP, and age); level of satisfaction of parents and physicians (5-point Likert scales).	Median pain scores: group I 4 (3–5), group II 2 (0–5), group III 0 (0–0), group IV 6 (4.6–9) Pain level ≤ 2: **group III 100%**, group II 57.6%, group I 25%, group IV 0% (*p* < 0.001). Parent satisfaction: **group III higher** compared to all other groups. Mean number of injections: **group III 7.8 ± 2.1**, groups I 5.2 ± 2.5, group II 5.7 ± 2.3, group IV 5.6 ± 3.3. **Pain was inversely correlated with age.**	/
Ben-Pazi H [33] 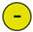	Quasi-randomized crossover trial	45 (31 M,14 F); mean age 7.04 y ± 4.68 y	CP: 16 DP, 13 HP, 15 QP, 1 TrP; GMFCS 2.86 (1.07)	Clown care vs. standard care	Expected pain and post-procedural pain measured by means of VAS: FPS (range 1–5) for children or VAS for parent.	Mean VAS-after 1st: **clown care 2.89 ± 1.36**, controls 3.85 ± 1.39. Mean VAS-after 2nd (carryover effect): **clown care 2.84 ± 1.38**, controls 4.11 ± 0.93. Mean difference between experienced pain and expected pain: clown care −0.34 ± 1.58, controls +0.74 ± 1.78. **Younger children reported higher pain**.	/
Ostojic K [34] 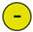	Crossover RCT	38 (20 M,18 F), mean age 13 y 5 mo ± 3 y 4 mo; 10 dropouts	34 CP; 4 HSP or ABI	Biofeedback-assisted relaxation training (BART) vs. distraction therapy	Mean and worst pain by means of FPS (range 0–10). Fear by means of Children’s Fear Scale (range 0–4). Anxiety by means of state–trait Anxiety inventory (range 0–60).	No significant difference in mean and worst pain, fear, and anxiety.	/

Legend: ABI, acquired brain injury; BART, biofeedback-assisted relaxation training; BoNTA, botulinum neurotoxin-A; CP, cerebral palsy; DP, diplegic; F, females; FLACC, Face Legs Activity Cry Consolability; FPS, faces pain scale; GMFCS, gross motor function classification system; h, hours; HP, hemiplegic; HSP, hereditary spastic paraplegia; iv, intravenous; M, males; mo, months; N₂O, inhaled nitrous oxide; QP, quadriplegic; TrP, triplegic; VAS, visual analogue scale; WBS, Wong–Baker faces pain rating scale; y, years. * age and results are reported in terms of mean values ± SD or (range) or median values (IQR); ^#^ statistically significant results are in bold. The “Traffic Light” tab represents the overall judgment based on the RoB assessment by means of RoB2 or ROBINS-I tools: red tab with exclamation mark, critical; red crossed tab, serious or high risk; yellow tab, moderate risk or some concerns; green tab, low risk.

**Table 2:** Characteristics of included case series.

Reference	Study Type	Population *	Diagnosis	Procedures	Intervention	Outcome	Results *^#^	Side Effects
Brochard S [35] 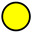	Prospective study	34 (18 M, 16 F); mean age 5,94 y ±4.21 y	33 CP: 10 HP, 12 DP, 11 QP; 1 adductor hypertonus relating to an orthopedic problem	209	N₂O and Emla^®^	Maximal pain over the whole procedure by means of CHEOPS (range 4–13). Pain during localize-by-electrostimulation, puncture, and injection phases. Pain assessed 30’ after the procedure by means of VAS for parents and children ≥ 6 y or FPS for children < 6 y (range 0–10).	Mean maximal CHEOPS 8.50 ± 3.56. **Mean CHEOPS during injection 7.40 ± 3.56 > localize 6.24 ± 3.07 > puncture 5.3 ± 2.44**. Mean children VAS/FPS 2.41 ±2.51 in 24/51 sessions. Mean parents VAS 3.59 ±2.58 in 38/51 sessions. No correlation between max CHEOPS and age.	3 children vomited after the session with no other complications; 2 children had particularly vivid dreams.
Gubbay A [36] 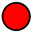	Comparative study (1st audit)	105 (72 M, 33 F); mean age 7 y 1 mo ± 3 y 11 mo	CP	105	Oral midazolam (0.5 mg/kg, max dose 12 mg) vs. MIKE (midazolam 0.5 mg/kg ketamine 0.3 mg/kg orally) in 18 patients	Parents satisfaction.	Oral midazolam: 89.5% tolerated the procedure well; 85% satisfied. MIKE: 67% tolerated the procedure adequately; of 11 children who had previously undergone BoNT-A with midazolam alone, 8 parents reported that the procedure was better tolerated when using MIKE versus midazolam alone.	1 transient distress after MIKE procedure.
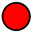	Comparative study (2nd audit)	108 children (62 M, 46 F); mean age 8 y 11 mo ± 3 y 11 mo	CP: GMFCS I 63, II 30, III 15; majority spastic DP	108	Intranasal fentanyl (1.5 µg/kg, max dose 75 µg) and topical anesthetic cream vs. 1st audit sedation methods	Effectiveness reported by parents and safety.	75% parents reported that intranasal fentanyl provided superior analgesia compared to previous methods (more effective in children weighing over 20 kg).	No serious adverse events.
Brochard S [37] 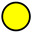	Monocentric prospective study	50 (31 M, 19 F); mean age 6.6 y ± 4.32 y ^§^	CP: 18 DP, 20 HP, 12 QP; GMFCS: 10 I, 20 II, 6 III, 10 IV, 4 V	199	N₂O (50% nitrous oxide/oxygen) and Emla^®^	Maximal pain over the whole procedure (CHEOPS). Pain during localize-by-electrostimulation, puncture, and injection phases. Pain in injected muscle.	Mean maximal CHEOPS 8.16 ± 3.5 (38% CHEOPS > 9). **Mean CHEOPS during injection (6.77 ± 3.30) > localization (5.46 ± 2.03) > puncture (4.88 ± 2.03).** **CHEOPS in adductors** < gastrocnemius.	^§^ 3 children vomited after the session with no other complications; 2 children had particularly vivid dreams.
Forrester M [38] 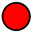	Prospective observational cross-sectional audit	171 CP; mean age 7 y 1 mo	CP	171	N₂O and topical anesthetic (conscious sedation) vs. GA	Carer perception of their child’s pain (no or slight, moderate, severe to extreme) and satisfaction. Median number of staff required.	**Perception under conscious sedation: 78% no or slight, 14% moderate, 8% severe to extreme >** under GA: 98% no or slight, 2% severe to extreme. Overall satisfaction: no sign. diff. **Median number of staff required: conscious sedation 3 <** GA 4.	Minimal adverse events.
Nilsson S [39] 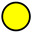	Retrospective study	61 (36 M, 25 F); mean age 5.7 y (1.4–13.8 y)	16 USCP, 38 BSCP, 7 DCP. GMFCS I 25, II 5, III 8, IV 11, V 12.	128	Rectal midazolam (mean dose 0.26 mg/kg, range 0.15–0.37 mg/kg) and racemic ketamine (mean dose 3.9 mg/kg, range 2.50–4.90 mg/kg) + Emla^®^ and oral or rectal paracetamol (30 mg/kg) 1 h before injection	Pain by means of FLACC for nurse and CAS (0–10) for parents. Feasibility (0–10 scale). Total time spent in the clinic. Frequency of side effects in 24 h (questionnaire for parents).	Median FLACC 2.0 (0–8): 85% 1–3; 10.8% 4–5; 4.2% > 5. Median CAS 2.0 (0–10). Median feasibility 9 (6–10). Median time 3.25 h (1.5–6.0 h).	Nausea 21.7%; pain 8.9%; sleep disturbance 7.7%.
Chow C [40] 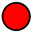	Retrospective study	87 (46 M, 41 F); median age 5 y 5 mo (range: 1 y 5 mo −13 y 2 mo)	CP: GMFCS II 52, III 21, IV 8, V 6.	152	Topical anesthetic cream, iv ketamine (median dose 1.0 mg/kg, range 0.7−2.2 mg/kg), and/or iv midazolam (median dose 0.1 mg/kg, range 0.06−0.45 mg/kg); In 92.1% procedures, iv atropine to reduce airway secretions	Efficacy: frequency of successfully completed procedures. Safety: frequency of adverse events at 2–3 weeks post-procedure follow-up review.	100% (152) successfully completed procedures. 6.6% were associated with mild, self-limiting adverse events. See box on the right.	4 rashes, 3 nausea and vomiting, 1 limb tremors, 1 mild headache, 1 nightmare in the post-procedure night. No serious adverse events.
Mondon-Willaume A [41] 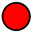	Prospective study	29; mean age 6 y 8 mo	CP and spastic children	30	N₂O, topical anesthetic, systematic analgesia with more or less sedative drugs	Maximal pain over the whole procedure (puncture, stimulation for localizing, injection) by means of FLACC (range 0–10). Percentage of patients with FLACC max < 3/10 (controlled pain group).	Mean max FLACC 2.10 ± 2.45. Controlled pain group: 65.5% (FLACC max < 3/10). Painful group (4 y 9 mo) significantly younger than controlled pain group (7 y 8 mo).	/
Fisher MT [42] 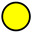	Retrospective study	249; mean age 9.2 y ± 5.6 y	189 CP, 60 other neurologic and musculoskeletal conditions	563	Vapocoolant spray vs. no vapocoolant, and in a 11 procedures topical anesthetic, and in 6 procedures, oral sedative	Pain by means of FPS (0–10) for the child or the parent during and after the procedure. Regression analysis to determine predictors of pain during and post procedure.	Mean FPS: overall 3.8 ± 3.0; vapocoolant spray: 3.9 ± 3.0; no vapocoolant 3.1 ± 2.7. Predictors of procedural pain: topical anesthetic; leg, hand, thigh injections; younger age. Predictors of post-procedural pain: pain during the procedure; older age.	/
Chau B [43] 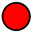	Retrospective study	14 (5 M, 9 F); mean age 7.79 y ± 2.39 y	12 CP median GMFCS * 2.25 (1.38–4), 1 spinal cord injury, 1 pontine hemorrhage	14	Individually set VR experience by means of VR headset and VR-capable smartphone, from publicly-available 360° videos via YouTube	Pain (FLACC). Caregiver feedback: positive, neutral, or negative experience with VR.	9 positive: median FLACC 2.5 (1 patient NR); 2 neutral: median FLACC 5.5; 2 negative: median FLACC 9.5; 1 unable to complete.	No adverse events.
Louer, R. [44] 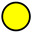	Retrospective study	164 (102 M,62 F); median age 9 y (4–11)	CP, GMFCS I 1, II 21, III 50, IV 55, V 33	345	Propofol and ketamine	Frequency of adverse events. Median procedure time, recovery time, total sedation time.	Adverse events: overall, 10.1% of procedures; all episodes were transient and resolved with supplemental oxygen. See box on the right. Median procedure time 10’, recovery time 11’, total sedation time 33’.	Hypoxemia 9.6%; transient apnea 1.4%; 0.9% hypoxemia and transient apnea. No serious adverse events.
Houx, L [45] 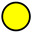	Prospective observational study	59 (35 M, 24 F); median age clowns 8 y (5–10) vs. controls 7.5 y (5–11)	52 CP, 12 cognitive disorders.	88	N₂O and Emla^®^ + medical clowns vs. usual distractions (music, television, video games as controls)	Pain: FLACC and VAS for the child and parent. Anxiety: VAS for the child and parent(s). Success of the sessions: 4-point Likert scale for physician and parents. Benefits of the distraction: 4-point Likert scale for parents.	Median maximal FLACC: clowns 2.5 (1–4) vs. controls 3 (1–4.3). Median VAS self-reported: clowns 2.5 (0–5) vs. controls 3 (1–6.3). Median VAS proxy: clowns 2.5 (0.3–3.4) vs. controls 3 (1–4.5). Median Likert success for parents: clowns 4 (4–4) vs. controls 3 (4–4) Median Likert benefits: clowns 4 (4–4) vs. controls 4 (3–4).	/

Legend: BSCP, bilateral spastic CP; CAS, colored analogue scale; CHEOPS, Children’s Hospital of Eastern Ontario Pain Scale; CLS, child life specialist; CP, cerebral palsy; DCP, dyskinetic CP;DP, diplegic; F, females; FLACC, Face Legs Activity Cry Consolability; FPS, faces pain scale; GA, general anesthesia; GMFCS, gross motor function classification system; h, hours; HP, hemiplegic; iv, intravenous; M, males; mo, months; MIKE, midazolam and ketamine oral protocol; N₂O, inhaled nitrous oxide; QP, quadriplegic; SA, sedation analgesia; TrP, triplegic; USCP, unilateral spastic CP; VAS, visual analogue scale; VR, virtual reality; WBS, Wong–Baker faces pain rating Scale; y, years. * age, characteristics of population and results are reported in terms of mean values ± SD or (range) or median values (IQR); ^§^ included 26 patients from Brochard et al. [36]; ^#^ statistically significant results are bolded. The “Traffic Light” tab represents the overall judgment based on the quality assessment by means of JBI tool: red tab, critical; yellow tab, fair.

## Data Availability

The original contributions presented in this study are included in the article/Supplementary Material. Further inquiries can be directed to the corresponding author.

## Supplementary Materials

The following supporting information can be downloaded at: https://www.mdpi.com/article/10.3390/toxins17070317/s1, Figure S1: Risk of bias assessment of included crossover studies by means of the RoB2 tool; Figure S2: Risk of bias assessment of included IRPG (parallel group) RCT by means of the RoB2 tool; Figure S3: Risk of bias assessment of included non-randomized controlled studies by means of the ROBINS-I tool; Table S1: Risk of bias assessment of included case series by means of the Joann Briggs Institute (JBI) tool; Table S2: Search strategy on the databases for PICO 1 and 2.
