# Supplementary material for: Procedural Pain Management in Patients with Cerebral Palsy Undergoing Botulinum Toxin Injection: A Systematic Review and Meta-Analysis
Source: Toxins (Basel). 2025 Jun 22;17(7):317. doi: 10.3390/toxins17070317 (PMC12298486; doi:10.3390/toxins17070317)
Supplement: Supplementary file 1 [file toxins-17-00317-s001.zip › Table S1.pdf]

Table S1. Risk of bias assessment of included Case Series by means of Joann Briggs Institute (JBI) tool.

	Brochard S 2009	Brochard S 2011	Fisher MT 2018	Houx L 2020	Nilsson S 2017	Gubbay A 2009 1st	Gubbay A 2009 2nd	Mondon-Willaume A 2017	Chau B 2018	Chow C 2016	Louer R 2019	Forrester M 2012
1. Were there clear criteria for inclusion in the case series?	Y	Y	U	U	U	N	N	N	N	N	Y	N
2. Was the condition measured in a standard, reliable way for all participants included in the case series?	Y	Y	Y	Y	Y	Y	Y	Y	Y	Y	Y	Y
3. Were valid methods used for identification of the condition for all participants included in the case series?	Y	Y	Y	Y	Y	Y	Y	Y	Y	Y	Y	N
4. Did the case series have consecutive inclusion of participants?	N	U	U	N	Y	U	U	Y	U	U	U	U
5. Did the case series have complete inclusion of participants?	U	U	U	U	U	U	U	U	U	U	U	U
6. Was there clear reporting of the demographics of the participants in the study?	Y	Y	Y	Y	Y	Y	N	U	Y	Y	Y	U
7. Was there clear reporting of clinical information of the participants?	Y	Y	Y	Y	Y	Y	N	N	Y	Y	Y	N
8. Were the outcomes or follow-up results of cases clearly reported?	Y	Y	Y	Y	Y	Y	Y	Y	Y	Y	Y	Y
9. Was there clear reporting of the presenting sites/clinics' demographic information?	Y	Y	Y	Y	Y	Y	Y	Y	Y	Y	Y	U
10. Was statistical analysis appropriate?	Y	Y	Y	Y	Y	U	U	NA	U	NA	Y	U
Overall	8	8	7	7	7	6	4	5	6	6	8	2
Judgement	Fair	Fair	Fair	Fair	Fair	Critical	Critical	Critical	Critical	Critical	Fair	Critical

Legend: 1. Were there clear criteria for inclusion in the case series?; 2. Was the condition measured in a standard, reliable way for all participants included in the case series?; 3. Were valid methods used for identification of the condition for all participants included in the case series?; 4. Did the case series have consecutive inclusion of participants?; 5. Did the case series have complete inclusion of participants?; 6. Was there clear reporting of the demographics of the participants in the study?; 7. Was there clear reporting of clinical information of the participants?; 8. Were the outcomes or follow up results of cases clearly reported?; 9. Was there clear reporting of the presenting site(s)/clinic(s) demographic information?; 10. Was statistical analysis appropriate?; Y, yes; N, no; U, unspecified; NA, not applicable.
