# Supplementary material for: Procedural Pain Management in Patients with Cerebral Palsy Undergoing Botulinum Toxin Injection: A Systematic Review and Meta-Analysis
Source: Toxins (Basel). 2025 Jun 22;17(7):317. doi: 10.3390/toxins17070317 (PMC12298486; doi:10.3390/toxins17070317)
Supplement: Supplementary file 1 [file toxins-17-00317-s001.zip › Table S2.pdf]

Table S2. Search strategy on the databases for PICO 1 and 2.

PICO 1	Database	Date	Strategy	Filters	Results
	Pubmed	12/02/2024	(botulinum toxins [MeSH Terms] OR cerebral palsy [MeSH Terms]) AND ((pain, procedural [MeSH Terms] OR (pain [MeSH Terms] AND prevention and control [MeSH Subheading])) OR analgesia [MeSH Terms] OR sedation OR nitrous oxide [MeSH Terms])	English<br>Humans<br>No meta-analysis<br>No reviews	279
	Cinahl	12/03/2024	(cerebral palsy or cp or spastic quadriplegia or spastic quadriplegic cerebral palsy) AND (analgesia or pain relief or pain management) AND botulinum toxin AND (procedural pain or painful procedures or procedure related pain or pain management or treatment related pain or invasive procedures)	English<br>Humans<br>No meta-analysis<br>No reviews	69
	Scopus	12/05/2024	(TITLE-ABS-KEY(botulinum toxin) AND TITLE-ABS-KEY(procedural AND pain OR analgesia OR sedation) AND TITLE-ABS-KEY(cerebral palsy))	English<br>Humans<br>No meta-analysis<br>No reviews	14
PICO 2	Pubmed	12/16/2024	(botulinum toxins [MeSH Terms] OR cerebral palsy [MeSH Terms]) AND (analgesia [MeSH Terms] OR sedation) AND adverse effects [MeSH Subheading]	English<br>Humans<br>No meta-analysis<br>No reviews	41
	Cinahl	12/17/2024	(cerebral palsy or cp or spastic quadriplegia or spastic quadriplegic cerebral palsy) AND (analgesia or pain relief or pain management) AND botulinum toxin AND (procedural pain or painful procedures or procedure related pain or pain management or treatment related pain or invasive procedures) AND (adverse effects OR advers events)	English<br>Humans<br>No meta-analysis<br>No reviews	10
	Scopus	12/18/2024	(TITLE-ABS-KEY(botulinum toxin) AND TITLE-ABS-KEY(procedural AND pain OR analgesia OR sedation) AND TITLE-ABS-KEY(cerebral palsy) AND TITLE-ABS-KEY (adverse AND effects OR adverse AND events))	English<br>Humans<br>No meta-analysis<br>No reviews	7
