# Supplementary figures and images for: Procedural Pain Management in Patients with Cerebral Palsy Undergoing Botulinum Toxin Injection: A Systematic Review and Meta-Analysis
Source: Toxins (Basel). 2025 Jun 22;17(7):317. doi: 10.3390/toxins17070317 (PMC12298486; doi:10.3390/toxins17070317)

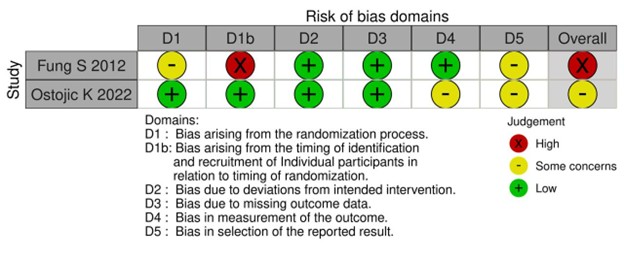

Supplement: Supplementary file 1 [file toxins-17-00317-s001.zip › Figure S1.jpg]

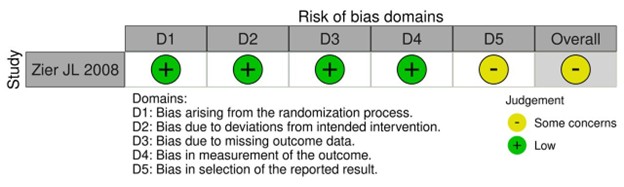

Supplement: Supplementary file 1 [file toxins-17-00317-s001.zip › Figure S2.jpg]

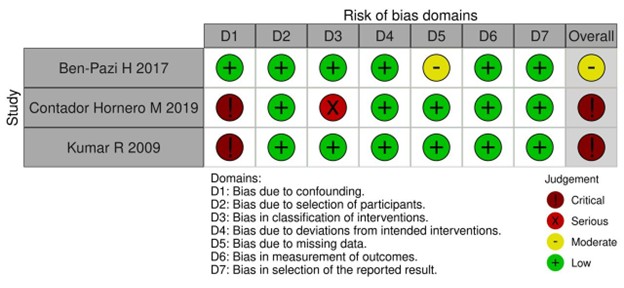

Supplement: Supplementary file 1 [file toxins-17-00317-s001.zip › Figure S3.jpg]
